# Bioaccessibility and bioactive potential of different phytochemical classes from nutraceuticals and functional foods

**DOI:** 10.3389/fnut.2023.1184535

**Published:** 2023-07-27

**Authors:** Alexandru Nicolescu, Mihai Babotă, Lillian Barros, Gabriele Rocchetti, Luigi Lucini, Corneliu Tanase, Andrei Mocan, Claudiu I. Bunea, Gianina Crișan

**Affiliations:** ^1^Department of Pharmaceutical Botany, “Iuliu Hațieganu” University of Medicine and Pharmacy, Cluj-Napoca, Romania; ^2^Laboratory of Chromatography, Institute of Advanced Horticulture Research of Transylvania, University of Agricultural Sciences and Veterinary Medicine, Cluj-Napoca, Romania; ^3^Department of Pharmaceutical Botany, Faculty of Pharmacy, “George Emil Palade” University of Medicine, Pharmacy, Sciences and Technology of Târgu Mures, Târgu Mures, Romania; ^4^Centro de Investigação de Montanha, Instituto Politécnico de Bragança, Bragança, Portugal; ^5^Laboratório Associado Para a Sustentabilidade e Tecnologia em Regiões de Montanha (SusTEC), Instituto Politécnico de Bragança, Bragança, Portugal; ^6^Department of Animal Science, Food and Nutrition, Università Cattolica del Sacro Cuore, Piacenza, Italy; ^7^Department for Sustainable Food Process, Università Cattolica del Sacro Cuore, Piacenza, Italy; ^8^Viticulture and Oenology Department, Advanced Horticultural Research Institute of Transylvania, Faculty of Horticulture and Business in Rural Development, University of Agricultural Sciences and Veterinary Medicine Cluj-Napoca, Cluj-Napoca, Romania

**Keywords:** bioaccessibility, nutraceuticals, functional foods, phytonutrients, polyphenols, carotenoids

## Abstract

Nutraceuticals and functional foods are composed of especially complex matrices, with polyphenols, carotenoids, minerals, and vitamins, among others, being the main classes of phytochemicals involved in their bioactivities. Despite their wide use, further investigations are needed to certify the proper release of these phytochemicals into the gastrointestinal medium, where the bioaccessibility assay is one of the most frequently used method. The aim of this review was to gather and describe different methods that can be used to assess the bioaccessibility of nutraceuticals and functional foods, along with the most important factors that can impact this process. The link between simulated digestion testing of phytochemicals and their *in vitro* bioactivity is also discussed, with a special focus on the potential of developing nutraceuticals and functional foods from simple plant materials. The bioactive potential of certain classes of phytochemicals from nutraceuticals and functional foods is susceptible to different variations during the bioaccessibility assessment, with different factors contributing to this variability, namely the chemical composition and the nature of the matrix. Regardless of the high number of studies, the current methodology fails to assume correlations between bioaccessibility and bioactivity, and the findings of this review indicate a necessity for updated and standardized protocols.

## 1. Introduction

When talking about food sources that are capable of delivering certain health benefits, the terms “nutraceuticals” and “functional foods” emerge. A nutraceutical is usually considered a product that is formulated (in different dosage forms) and taken orally to promote health, and the term functional food is retained for any product with such properties, but consumed as food (1–4). Aluko, in turn, describes functional foods as products that look alike “conventional food,” but which are able to reduce certain chronic diseases, apart from being a source of useful nutrients (e.g., tomato fruit, oatmeal, fish, soybean, tea and sour milk). On the other hand, to obtain a “nutraceutical,” one has to isolate and purify phytocompounds from the food sources and formulate them accordingly (e.g., pills with isoflavones from soybean seeds, liquid extracts of plant origin and capsules containing fish oil) (5). For their properties, nutraceuticals have also been described as “medical foods” and dietary or nutritional supplements (3).

**Figure 1:**
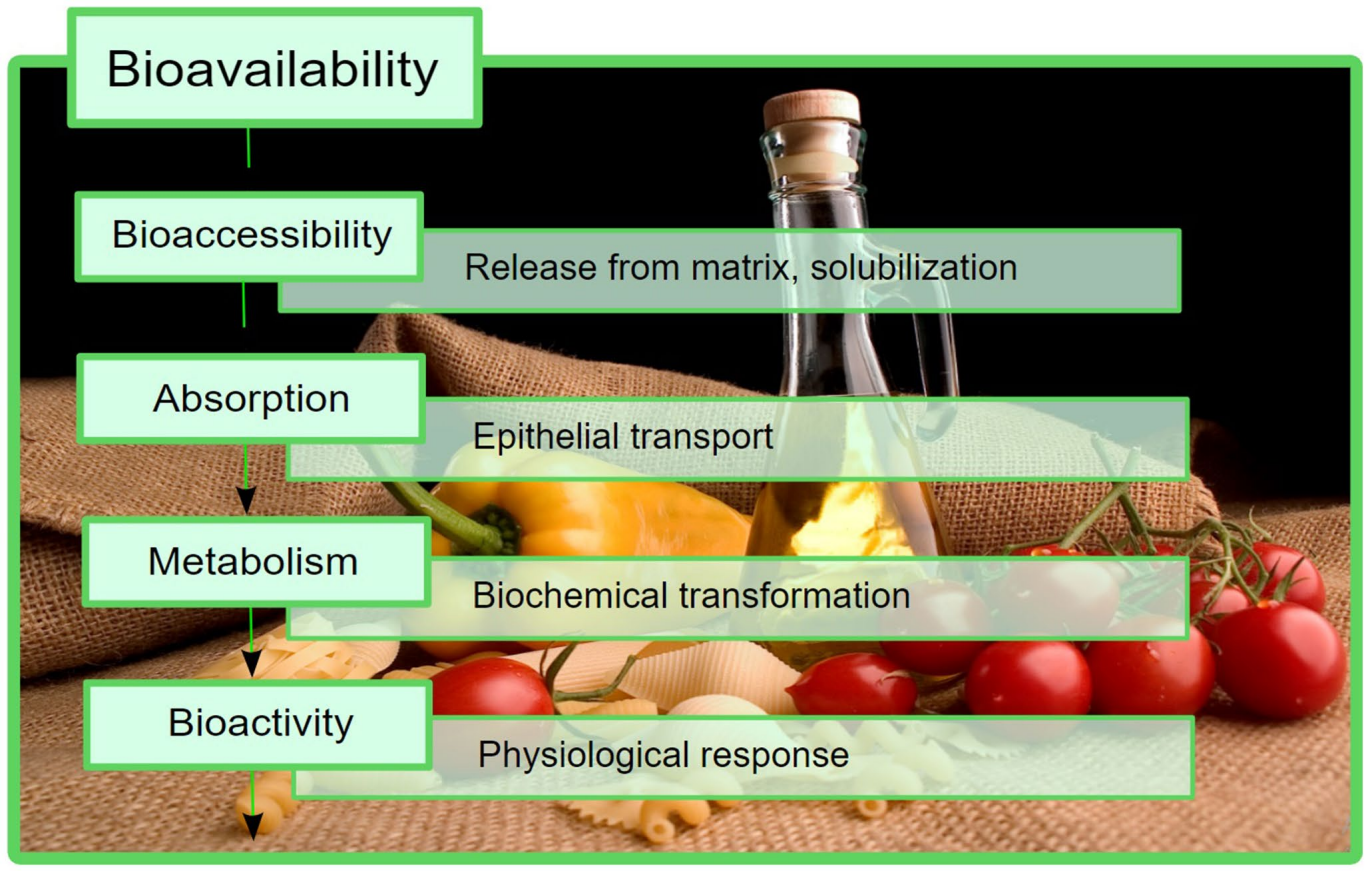
Bioavailability presented as a sum of different stages that take place *in vivo*. In this process, bioaccessibility is an important factor that should be considered.

In spite of the high composition of phytochemicals with beneficial effects, the use of the abovementioned products is limited by their bioavailability, which can be described as the fraction of a compound that reaches the biological target by distribution, after being absorbed from the gastrointestinal tract (GIT) into the circulatory system, i.e., it enters the blood stream (6, 7). The terms “bioaccessibility” and “bioactivity” were introduced to accurately describe the steps in which a compound becomes bioavailable. There are several suitable definitions for bioaccessibility, but the most comprehensive one describes it as the fraction of a compound that remains available for absorption at the intestinal level, subsequent to all of the physiological transformations that can occur locally, including the enzyme-mediated degradation (6, 8). Additionally, the term “bioactivity” is currently used to describe a physiological effect of a certain compound, specifically focusing on its interaction with the target biomolecules after distribution (6, 7). A schematic representation of how these processes constitute the final bioavailability is presented in Figure 1.

The magnitude of the bioavailability, specifically for compounds of plant origin, correlates with certain factors, the most important being the release of the nutrients from the matrix, the variability of physiological digestion conditions and the pharmacokinetics of the compounds, including epithelial absorption, biochemical degradation and distribution (6). Likewise, a very relevant factor for lipophilic compounds is the solubility and the possible lack of solubilization in the GIT (9), and a low permeability correlates with low bioavailability assets (6). In this case, virtual techniques can also assess the permeability and bioavailability of certain compounds (10).

To measure the bioavailability of a certain compound, complex and challenging *in vivo* methods are required, seeking to investigate its pharmacokinetic properties. On the other hand, the determination of bioaccessibility is frequently carried out with satisfactory results through *in vitro* methods, which aim to simulate the physiological conditions (including an accurate reproduction of the chemical and mechanical properties) inside the GIT. The recent methods described mention the simulation of oral, gastric and intestinal digestion, with the possibility of separating the small and large intestine into different sections (6, 7, 11, 12). The applicability of these methods covers a high number of classes of natural bioactive compounds, relevant examples being phenolic derivatives (13, 14), carotenoids (12, 15), and even biotechnologically-derived bioactive compounds (such as vitamins) (16, 17). Along the advances in formulation research, new classes of compounds are studies for their potential as highly bioaccessible nutraceuticals and functional foods, a peculiar example being bioactive peptides (18, 19).

When studying the therapeutic potential of natural products, including nutraceuticals, plant foods and phytochemical products, the determination of bioaccessibility seems of utmost importance, since it acts as an indicator of whether the initial compounds will maintain their bioactivity intact or lose it gradually under the influence of numerous factors. Therefore, these assays are capable of foreseeing the possible bioavailability that could be determined *in vivo* (12, 20). Consequently, the attempt of the present review was to establish the importance of bioaccessibility determination as a prediction tool for the bioavailability of phytonutrients in the specific case of nutraceuticals, nutritional supplements, and functional foods, using information available at the moment, and to additionally determine the possible influence of simulated digestion conditions on the bioactivity of certain phytochemicals. A review of examples of recent advances in the research on this topic is presented in Table 1. Unfortunately, despite the excessive research data in this field available in the literature, virtually no paper managed to achieve a general trend regarding the correlation between bioaccessibility determination and *in vitro* bioactivity, aside from unclear outcomes and suggestions for future research. Ultimately, this situation leads to an imperative necessity to (1): standardize the protocols and the techniques used in qualitative analysis (2); standardize the method for bioaccessibility expression, due to the tedious interpretation needed in the present research; and (3) interpret the results in order to show the correlation between simulated digestion and bioactivity assessment, and also between *in vitro* and *in vivo* behavior of compounds. We consider that these highlights represent the novelty element of the present critical review.

**Table 1:** Examples of research studies that aimed to determine a link between bioaccessibility testing (simulated digestion) and bioactivity in the case of functional foods and nutraceuticals or dietary supplements of plant origin.

No.	Nutraceutical/functional food and plant species	Determined bioaccessibility (or analogous methods of quantification) and bioactivity after simulated digestion	Effect of simulated digestion on bioactivity	References
	Phenolic compounds			
1.	Rehydrated wild and commercial blackberries (*Rubus* spp.)	Decrease % (after IP) 70% in wild and 68.35% in commercial berries (TPC) 74% in wild and 75% in commercial berries (TAC) 52% in wild and 56% in commercial berries (ORAC) 43.8% in wild and 41.6% in commercial berries (DPPH) 40.47% in wild and 46.73% in commercial berries (ABTS)	SD decreased TPC and TAC after IP SD decreased antioxidant activity (for ORAC, DPPH, ABTS), depending on the fruit sort	(21)
		Increase % (after IP) 56% in wild and 23% in commercial berries (Caco-2 cells cytotoxicity, as EC_50_)	SD decreased the cytotoxicity of samples after IP	
2.	Passion fruit peel 70% ethanolic extract (*Passiflora edulis*)	Recovery index (after IP) 97% (TPC), 84.4% (TFC), 25.8% (TAC) Decrease in activity (after IP) 32% (DPPH), 30% (FRAP) Increase in activity (after IP) 17% (ABTS)	SD decreased phenolics, flavonoids and anthocyanins SD decreased α-glucosidase inhibitory activity SD decreased the DPPH and FRAP antioxidant activity, but increased the ABTS scavenging activity	(22)
3.	Broccoli sprouts ethanolic extracts (*Brassica oleracea* L. var. *italica*)	Antioxidant BA index (after IP) 1.09 (lipid peroxidation inhibition), 0.39 (chelating power), 0.44 (ABTS), 1.75 (FRAP)	SD decreased the antioxidant effect No correlation between SD and antiproliferative effect	(23)
4.	Quinoa leaves ethanolic extracts (*Chenopodium quinoa*)	Antioxidant BA index (after IP) 1.52 (chelating power), 4.37 (reducing power), 1.32 (lipid peroxidation inhibition), 4.74 (ABTS), 0.41 (lipoxygenase inhibitory activity)	SD decreased the antioxidant effect, but increased lipoxygenase inhibitory activity	(24)
5.	Black mulberry jam (*Morus nigra*)	Increase in recovery (as %) Fruit: 12% (TPC), 1% (TMA), 14% (ABTS) Jam: 16% (TPC), 12% (TMA), 37% (ABTS)	SD decreased phenolics and antioxidant activity Jam processing had a higher recovery after SD compared to raw fruits	(25)
6.	Fennel waste capsules (*Foeniculum vulgare*)	Acid resistant capsules – BA* 23.84% (TPC, DP), 63,14% (TPC, CP) 35.21% (DPPH, CP), 47.46% (ABTS, CP), 62.60% (FRAP, CP)	SD decreased TPC and antioxidant activity, but the highest values in SD were for CP During SD, acid-resistant capsules provided a higher antioxidant effect due to a higher polyphenol content	(26)
		Non-acid resistant capsules – BA* 20,92% (TPC, DP), 42.58% (TPC, CP) 26.06% (DPPH, CP), 36.72% (ABTS, CP), 43.90% (FRAP, CP)		
7.	Encapsulated lyophilized powder from tea extracts (acid-resistant capsules) (*Camellia sinensis*)	Green tea - BA 23.77% for DP and 108.85% for CP (total phenolics)	CP showed a higher antioxidant effect and TP DP showed a significantly lower antioxidant effect and TP	(27)
		White tea - BA 19.33% for DP and 102.21% for CP (total phenolics)		
		Black tea - BA 13.00% for DP and 112.26% for CP (total phenolics)		
8.	Health supplement mix with tart cherry extract and mineral clay (*Prunus cerasus*)	BA 109.5% for GP and 26.7% for DP (anthocyanins) 115.4% for GP and 177.1% for DP (ORAC)	SD decreased anthocyanin content, especially after DP SD increased antioxidant activity (ORAC), possibly because of other classes of bioactive compounds	(28)
	Carotenoids			
9.	Encapsulated carotenoids from red pepper waste (*Capsicum annuum*)	Freeze-dried encapsulates – BA 20.4% after SD (for total carotenoids) 40.24% for GP and 50.51% for IP (BCB)	SD decreased total carotenoids, higher BA for freeze-dried encapsulates SD increased antioxidant activity of carotenoids compared to GP	(29)
		Spray-dried encapsulates - BA 15,05% after SD (for total carotenoids) 31.56% for GP and 55.53% for IP (BCB)		
10.	Powders obtained from seed-used pumpkin byproducts (*Cucurbita maxima*)	18 mesh-sized powder - BA (after IP, relative to GP) 12.67% (total carotenoid relative BA) 35.8% (DPPH)*, 75% (FRAP)*	SD decreased total carotenoid relative BA and the antioxidant activity Increased antioxidant activity when 2% corn oil was used (for DPPH and FRAP)	(30)
		18 mesh-sized powder and 2% corn oil- BA (after IP, relative to GP) 27.0% (total carotenoid relative BA) 64.1% (DPPH)*, 95.7% (FRAP)*		
11.	Methanolic extracts of dried and juiced black plum (*Syzygium caryophyllatum*)	Fresh fruit – BA (after IP) 9.75% (β-carotene), 5.11% (lycopene), 15.29% (TPC), 33.65% (TFC) 4.55% (TACap)*, 61.42% (ABTS)*	SD decreased β-carotene and lycopene content, TPC and TFC SD decreased the antioxidant activity (TACap and ABTS)	(31)
		Dried fruit – BA (after IP) 43.53% (β-carotene), 9.48% (lycopene), 7.30% (TPC), 14.36% (TFC) 3,86% (TACap)*, 81.66% (ABTS)*		
		Juice – BA (after IP) 35% (β-carotene), 10.39% (lycopene), 7.37% (TPC), 21,69% (TFC) 2.55% (TACap)*, 33.49% (ABTS)*		

BA, bioaccessibility; SD, simulated digestion; GP, gastric phase; IP, intestinal phase; CP, colon phase; DP, duodenal phase; UD, undigested; BCB, beta-carotene bleaching assay; TAC, total anthocyanin content; TACap, total antioxidant capacity; TFC, total flavonoid content; TPC, total phenolic content; TMA, total monomeric anthocyanins.

^*^The values have been calculated using data from the article, if the final bioaccessibility value was not available.

## 2. Recent methods for determination and measurement of bioaccessibility

To ascertain the behavior of compounds inside the human organism, scientists have developed a vast number of useful techniques. Unfortunately, deciphering and foreseeing the exact processes that can take place is challenging, given the complexity that can arise (7). Initially, the best way to perform such tests was accomplished only through *in vivo* determinations, which were considered the most effective in describing the complexity of pharmacokinetic processes, i.e., absorption, distribution, metabolism and excretion, in parallel with toxicity studies (7, 32). Obviously, as one might expect, *in vivo* studies have some disadvantages, mainly the high cost, the low reproducibility and the difference between animal and human organisms, caused by metabolic disparities, which can alter the interpretation of results (6, 7).

To simplify the methodology, a few *in vitro* determination methods have been developed. The final aim is to ascertain the bioaccessibility of phytocompounds, i.e., to estimate the remaining fraction of these compounds available for the intestinal absorption (11, 32). An idealized *in vitro* method is one that is able to provide results with enough accuracy, in a short amount of time. However, any of the possible method available will be fundamentally unsuccessful in accurately describing the complexity of processes that take place in a living organism, reason why, in the last decade, there was a need to compromise between the comfort of simple methods and the accuracy of *in vivo* methods by establishing proper *in vitro* applications (33). During the last decades, a great variety of *in vitro* digestion methods have been developed, especially for food research. Regardless of the type of determination, all of these methods seek to simulate the physiological conditions that are present in the GIT, specifically for oral, gastric and intestinal digestion (34). One category of approaches is represented by static methods realized by fixing the concentration of the chemicals used for simulation, such as enzymes and bile salts, which complexity can be enhanced by using dynamic methods (in which a variation in concentration is desirable), simulating the variation of physiological conditions (35).

Recently, dynamic methods have also been greatly developed. For these, the control of the process is accomplished using computers (which includes the automatic adjusting of pH levels and the introduction of enzymes and food probes), with their main advantage being the possibility of a superior mimicking of the complexity of gastrointestinal processes (7, 35, 36). However, despite their superiority when compared to static methods, the dynamic ones could require a validation in relation to different types of matrices, especially regarding dosage forms. Therefore, to further improve these methods, several recent upgrades have been accomplished, e.g., using a mastication simulator for solid foods and coupling different types of cellular models (including CaCo-2 and HT-29 cells). Considering the necessity to continuously develop these techniques, future models might include the microbiota present in the small intestine (36). For example, Pérez-Burillo et al. (37) have recently developed a method that can be used after *in vitro* digestion, which improves its complexity by using a simulated fermenting microbiota composed of a peptonized fermentation medium and gut microbes originating in human feces. Considering all these factors, the most accurate method of testing bioaccessibility *in vitro* would be based on dynamic methods, considering all the phases of digestion and coupled with cellular models (38).

Even though a great effort is devoted to the quantitative analysis of phytochemicals in plant matrixes, it is worth mentioning that predicting their bioavailability is far more important than classical quantification. As more modern methods start to develop, it is important to analyze the preliminary stability of phytocompounds by digestive simulation, since this affects directly the existence of the potential biological effects that have been determined by *in vitro* assays (11, 32). In the case of nutraceuticals, *in vitro* digestion methods seem to be especially useful for evaluating their behavior, leading to the possibility of determining certain factors that can alter the final bioavailability, specifically when considering the degradation processes and bioaccessibility values (7). These studies are extremely relevant in food science research, due to the increasing necessity to obtain optimized, yet simple, supplement formulations that can be used in the prophylaxis and treatment of diseases (39).

### 2.1. Nutraceuticals and functional foods

As for nutraceuticals, which are most frequently taken orally, there are some factors that can alter their oral bioavailability. One of the most important factors is their bioaccessibility, commonly determined through *in vitro* methods, which aim to reduce the necessity of using high-cost and time-consuming *in vivo* pharmacokinetic studies. Furthermore, to predict their bioavailability with precision, there is a need for using more complex methods (by studying additional factors), and to obtain a good correlation degree between *in vitro* and *in vivo* studies (40). Both in dosage forms and as foods that contain a higher content of bioactive compounds, nutraceuticals are susceptible to variations, given the existence of external and internal factors that may influence their final bioavailability (41). For the process of fortification of functional foods with different compounds with known bioactivity, bioaccessibility testing is crucial, and future research should focus on validating the correlation between *in vitro* and *in vivo* studies to confirm the reliability of the former method (38, 42). Validation of *in vitro-in vivo* correlations is still under development, and further relevant data is needed to standardize this approach. Wu and Chen recently described some critical aspects necessary for this validation, as essential part in the rational development of functional foods; future studies should take into account: the establishment of a clear definition of bioaccessibility and validation, the construction of more realistic simulation systems, as well as the specification of prospective acceptability criteria. Such criteria should be based on the standards of the pharmaceutical industry, for example through FDA guidance (38).

An essential aspect related to functional foods is biological acceptability, since they represent a particular class of nutritional products with health-promoting qualities. In this case, consumer opinion and acceptability should be taken into account, and future bioaccessibility testing should be coupled with consumer tests, aiming to determine liking parameters such as: taste, aroma, odor, texture, flavor, and purchase predisposition (43–45).

Since nutraceuticals usually contain a mix of compounds with great variation regarding the chemical structure, their bioaccessibility and bioavailability are susceptible to variations according to certain chemical and physio-chemical parameters. When assessing bioaccessibility, there is the possibility of modulating different parameters to determine the oral efficiency, e.g., the pH and temperature ranges, and the enzyme activity. However, the kinetics of the absorption process can also be affected by the presence of other foods and not only by the aforementioned factors (40, 46). In favor of the absorption process, the bioactive phytochemicals from nutraceuticals must be liberated from the dosage form and consequently solubilized in the gastrointestinal fluid. The solubilization process depends strictly on the local physiological parameters and the chemical properties of the phytocompound, similarly to any drug used therapeutically. For instance, lipolytic enzymes are indispensable for the solubilization of a lipophilic compound, but the solubilization process for hydrophilic compounds is predisposed to variation due to the change in pH value and ionic strength (41). Furthermore, for the majority of lipophilic phytochemicals, such as carotenoids, A, D, E and K vitamins and fatty acids with longer chains, bioaccessibility seems to be the rate-limiting step, due to the necessity of forming mixed micelles for solubilization (47).

Moreover, the importance of bioaccessibility in determining the bioavailability of nutraceuticals and functional foods is illustrated by the influence of the so-called external factors, which can be operated upon to enhance the nutritional value and the quality of these products. For example, worth mentioning influential external parameters are: (a) chemical and physical properties of the nutraceutical product, (b) the adoption of new delivery systems, and (c) the processing and storage conditions of the product (41). For nutraceuticals in particular, the dosage form can significantly modify the final bioavailability of the phytonutrients originating in the matrix, given that certain excipients might raise their accessibility to intestinal absorption. Examples include: propylene glycol solutions, phospholipid complexes, nanoparticles and various colloidal systems (40).

Predicting the behavior of a nutraceutical that holds a high range of phytochemicals (with different structures and characteristics) is difficult, given that every individual physical and chemical property has to be considered. Likewise, no compound is ideal in this regard, as hydrophobicity is correlated with lower solubility in GIT fluids, and consequently with a decreased bioaccessibility. However, a hydrophilic behavior is linked to a higher solubility, yet a lower permeability through the epithelial wall (41). For example, in the case of polyphenol esters and glycosides (i.e., the main forms found in plants and foods), their solubility and absorption are very low, and they can only be absorbed as aglycones after enzymatic hydrolysis (48), mostly achieved under the influence of bacterial enzymes (49).

Interestingly, Thakur et al. (6) have recently identified the influence of certain processing techniques on the bioaccessibility of phytonutrients from different functional foods. When it comes to polyphenolic compounds and carotenoids, cooking processes induce the rupture of the cell wall, which induces a higher release of these phytochemicals. Also, the enhancement of matrix release was mostly identified for dehydration, thermal processing, drying, frying and the addition of different oils and fats, compared to raw products. On the other hand, other modern non-thermal processing technologies (such as the usage of ultrasounds, pulsed electric fields and high pressure) have been cited as methods to increase bioaccessibility, in this case the enrichment in phytochemicals probably being caused by promoting cell membrane permeability. However, such technologies might also induce a higher viscosity in the medium, due to the release of fibers and pectin, possibly with negative impact on bioaccessibility (50). Depending on the phytochemical composition and the plant or food matrix, the aforementioned thermal and non-thermal processing technologies can either increase or decrease their bioaccessibility, therefore their levels being not only affected by the selected method, but also by pre-treatment steps and the nature of the compounds under investigation (51).

Another important factor that can interfere with the absorption process by lowering the bioaccessibility is the presence of antinutrients, which are compounds that act as absorption inhibitors. To this day, several studies have noted the negative impact of such compounds (for example phytates, polyphenols and dietary fibers) on mineral and micronutrient bioavailability (52). To improve the efficacy of nutraceuticals and pharmaceuticals, the so-called “excipient foods” can be used, illustrative examples being olive oil added to carotenes and pectin added to β-carotene emulsions (53), or olive oil emulsions that increase the bioaccessibility of lycopene from tomato pomace (54). The usage of this class of products consists in the fact that they can be co-administered orally presenting no bioactivity but promoting the release of compounds of interest from the matrix (53, 55).

Besides, the bioaccessibility of nutraceuticals and/or phytochemicals with hydrophobic characteristics can be improved by using different colloidal delivery systems, frequently through encapsulation methods (41, 47) or emulsions (56). In certain cases, encapsulation techniques can be also applied to hydrophilic nutraceuticals (41). For lipophilic compounds, the best methods involve the utilization of lipids, either as liquids or semisolids, in the form of nanoemulsions, microemulsions, nanoparticles, carriers or emulsifying delivery systems. Also, phospholipids can be used for the preparation of liposomes (41). Another useful modern method is nanoencapsulation, which can be achieved by employing biopolymeric nanocarriers. By reducing the size of the particles to such extent, there is a higher absorption through the GIT (41, 57). For certain chemicals, the enhancement of nutraceutical bioaccessibility has been extensively investigated in recent years. One example is curcumin, which has been formulated using different excipient emulsions (58), emulsion-based delivery systems (59), Pickering emulsions (60), organogel-based emulsions (61), and even used for the fortification of dairy products (62) or plant-based milk analogs (63).

## 3. Phenolic compounds

Among all classes of phytochemicals, (poly)-phenolic compounds are one of the most numerous in plants, playing a significant role in the quality of plant-based food products. In particular, a diet rich in phenolic compounds has been associated with various beneficial effects, such as reducing the risk of cardiovascular diseases, cancers, diabetes and degenerative diseases, due to the substantial antioxidant effect and anti-inflammatory properties linked to them (64–66).

**Figure 2:**
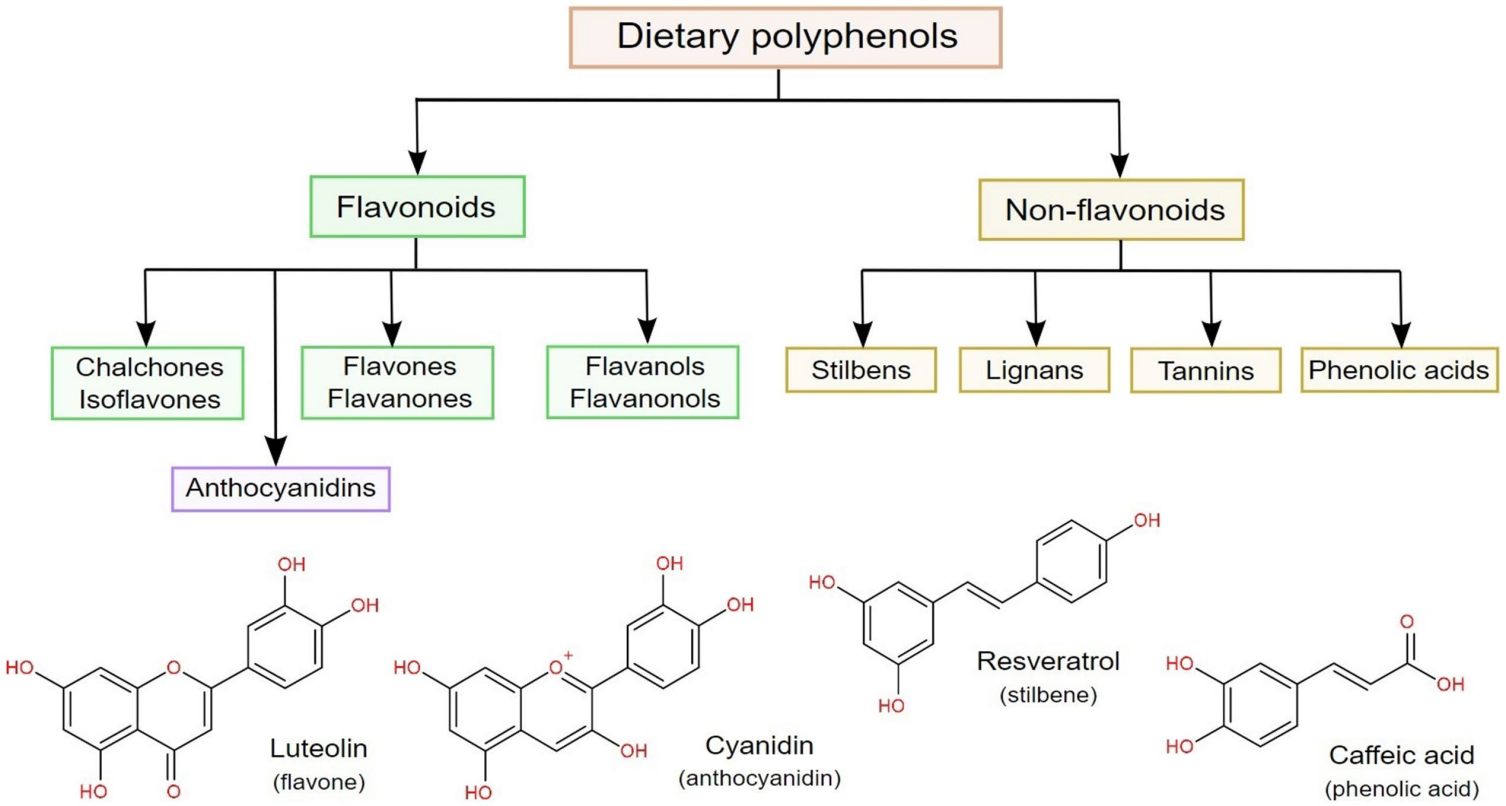
Classification of dietary polyphenols, along with the chemical structures of the most common representatives in four classes of polyphenols.

According to their chemical backbone, dietary polyphenols can be divided into flavonoids and non-flavonoids (6), as shown in Figure 2. From the latter, phenolic acids are the most common, being chemically identified as benzoic and cinnamic acid derivatives (with C6–C1 and C6–C3 structures, respectively), followed by stilbenes, lignans and tannins. On the other hand, flavonoids have a C6–C3–C6 general structure, and can be further subdivided according to hydroxylation patterns into flavan-3-ols, flavones, flavanones, flavonols and anthocyanidins (64, 67).

### 3.1. Bioaccessibility of phenolic compounds from nutraceuticals and functional foods

Polyphenolic compounds of natural origin can either or not be bound to different types of dietary fibers from the macromolecular matrix, which importantly affects their bioaccessibility (68). When polyphenols are linked to these fibers, an additional step is required to be released in the gut, being identified in lower quantities in comparison to unbound polyphenols (69). This insoluble fraction can also contain highly-polymerized phenolic species, such as tannins and lignins, which have a low bioaccessibility due to limited release (14). Presumably, there are certain factors that can lower polyphenols’ bioaccessibility, such as the presence of dietary fibers, minerals and high protein content. Nonetheless, some constituents could even enhance their accessibility, e.g., lipids (for lipophilic polyphenols) and digestible carbohydrates (69, 70). To increase the bioaccessibility of phenolic compounds from nutraceuticals or supplements, an appropriate formulation must be chosen. At the same time, modulating the composition and structural properties is important for optimizing functional foods. Recent studies observed the influence of the dosage form on the proper release during digestive simulation, with some of the relevant examples being the different encapsulation techniques (71–73), microencapsulation (74), fluid and gelled emulsions containing quercetin (75) and other lipid-based delivery systems, including nanoemulsions (76), lipid nanoparticles and liposomes (77). For nutraceuticals, developing new types of suitable encapsulation techniques (such as acid-resistant capsules) has the potential to improve the bioaccessibility of phenolics by preserving them (78).

One of the most important factors that influence the accessibility of flavonoids (the most abundant phenolic class) is their chemical structure and the presence of sugar moieties in their molecules, which play an important impact on their absorption, since only aglycones are accessible for this process. Also, the nature of the sugars is decisive, since glucosides are hydrolyzed by glucosidases (present especially in the small intestine) and other types of glycosides undergo different degradation pathways (6, 79). For instance, the bioaccessibility of some polyphenols is strongly affected by colonic biotransformation, which takes place under the influence of the gut microbiota where different metabolites are produced. In some cases, this degradation phase can positively affect the microbial populations by producing beneficial prebiotic metabolites (80). In part, the gastrointestinal biotransformation of polyphenols is decreased by the presence of certain micronutrients, such as vitamins C and E (70). Among the different classes of polyphenolic compounds, there are separate degradation patterns depending on the pH of the media, acidic or alkaline, and on the presence of enzymes that are capable of inducing hydroxylation, methylation and even glycosylation. These factors are especially important for anthocyanins, where the environment’s pH directly affects their antioxidant effect (81). In addition, formulating functional foods with anthocyanins can achieve a higher bioaccessibility by using optimal conditions, with regard to the food matrix composition, the extraction parameters, the chemical structure and the delivery system chosen, with a high concern for the pH (82).

The influence of the gut microbiota is significant in the colonic stage of digestion, where several chemical processes convert different classes of polyphenols into metabolites. For example, hydroxylation of the aromatic ring occurs frequently, and metabolites such as hydroxyphenylpropionic acid (e.g., from flavanols, flavones and flavanones) and hydroxyphenylacetic acid derivatives (e.g., from flavanols and ferulic acid dimers) can be found after this stage (81, 83). At the same time, anthocyanins degrade into a wide range of compounds, including benzoic acid, benzaldehyde and acetaldehyde derivatives (81), which can be absorbed much easier (84). Even though anthocyanins are generally stable during gastric digestion, they have a tendency to degrade at the intestinal level, and the chemical structure dictates their stability. In most cases, nonacylated anthocyanins present a weaker stability in comparison to acylated forms, 3,5-diglycosides being the most stable forms (85).

The bioaccessibility assessment of phenolic compounds can be achieved through classical methods, which are comprised by three gastric phases: oral stage (simulated with amylase at pH 7), gastric stage (simulated with pepsin at pH 2) and intestinal stage (simulated with pancreatin at pH 7). By all means, many parameters of the experimental model can be manipulated, including the concentration of bile extract and electrolytes, and temperature (14). Moreover, recent studies assessed the bioaccessibility of phenolic species using cell lines, usually models based on human cell carcinoma-derived Caco-2 cell line. This method is useful because the cells present a similar morphology to the cells of the small intestine, which allows the concurrent determination of the possible mechanisms of transport, including the permeability through the epithelial barrier (86–88). The experimental model implying Caco-2 cell lines has the advantage of being applicable to the assessment of cellular response and the absorption patterns of phenolics (89), and recent studies considered such models in order to predict the cellular uptake of certain phytocompounds (90, 91).

Subsequent to the simulation of the gastrointestinal digestion, the quantification can be realized through different methods. The most used alternatives are the centrifugation or the filtration of the digesta (for the determination of phenolics in supernatant), and the dialysis method (for the independent determination of high and low molecular weight compounds). The latter implies the usage of a dialysis tube, after the gastric or intestinal stage. Furthermore, the aforementioned dynamic methods are controlled by a computer and can be used as multi-compartmental models, with applicability to phenolics, vitamins and minerals (92). One of the most common methods is TNO-Intestinal Model (TIM), developed by Netherlands Organization for Applied Scientific Research. In TIM, the digestive simulation is applied through four compartments, corresponding specifically to gastric, duodenal, jejunal and ileal components, with the possibility of controlling the gastric emptying rate, the precise parameters of the peristaltic movement and the different chemical variations that take place *in vivo* (81). A recent paper by Santana et al. reviewed all the possible methods that can be used for the determination of polyphenols’ bioaccessibility from tropical fruits, including static, semi-dynamic and dialysis, transport or colonic fermentation methods (93).

### 3.2. Influence of bioaccessibility on the activity of phenolic compounds

A study by Cao et al. reported the reduction of total phenolics, flavonoids and anthocyanins after simulated digestion, which in turn caused a decrease in the bioactivity of passion fruit (*Passiflora edulis* hybrid) peel ethanol extracts. The alpha-glucosidase inhibitory activity seemed to be correlated with the anthocyanin content, which can explain the diminished effect caused by their low bioaccessibility (22). Some studies have shown that more polyphenols are extracted when there is a higher solvent-to-solid ratio. For grapes, Tagliazucchi et al. noted that there was a higher polyphenol extractability when a higher solvent-to-solid ratio was used, demonstrated by a rise in the antioxidant activity. Moreover, this bioactivity is significantly affected by the pH of the media and with the chemical processes that take place in the progressive digestion phases (94). Gawlik-Dziki et al. (24) investigated the phenolics in the ethanolic extract of *Chenopodium quinoa* leaves, showing a high antioxidant activity, an antiproliferative effect on prostate cancer cells, and a relatively high bioaccessibility. Thus, quinoa leaves could be taken into consideration as potential nutraceuticals. In another similar study, broccoli sprouts extracts showed a rise in phenolic compounds concentration after simulated gastrointestinal digestion. Still, the authors failed to find a correlation between these levels and the antiproliferative effect of the extracts on the AT-2 and MAT-Lylu cell lines (23).

Sánchez-Velázquez et al. compared the antioxidant activity of wild and commercial blackberries (*Rubus* spp.) after *in vitro* simulated digestion, concluding that wild berries exhibited higher bioaccessibility and antioxidant activity. Moreover, both sorts showed similar transformation patterns during digestion, and overall there was a reduction in total phenolic and flavonoid content, as well as in antioxidant activity (21). For black mulberry (*Morus nigra*) jam, the processing gives rise to a product that has a lower amount of total anthocyanins and polyphenols. At the same time, simulated digestion tests have shown that jam could provide a better matrix with a higher bioaccessibility for these compounds, in comparison to raw fruits, delivering a higher amount of antioxidant compounds (25). Zhang et al. (95) showed that there is a relatively high anthocyanin bioaccessibility for two purple root vegetables (44.62% for purple carrot and 71.8% for purple potato), possibly due to a higher resistance to gastrointestinal degradation. Bertolino et al. studied the possible fortification of yoghurt with bioactive compounds from coffee silverskin, concluding that this by-product could be used as a source of dietary fibers and phenolic compounds. Furthermore, the simulation of digestion induced an increase in the antioxidant activity of the yoghurt samples, which shows the possibility of a rise in bioaccessibility in this combination (96). On the other hand, Pereira-Caro et al. studied the effect of simulated digestion on two food products derived from black carrot (as snack and seasoning), in comparison to the vegetable alone, and observed that the oral digestion induced a significant decrease in antioxidant activity, with a smaller decrease in the following digestion stages. Regarding the analysis of the phenolic compounds, anthocyanins showed better bioaccessibility from the derived products and were affected by oral digestion, but hydroxycinnamic acid derivatives showed higher stability during digestion. The authors have also used fecal fermentation using human fecal microbiota, and observed a significant conversion of phenolic species, 3-(4′-hydroxyphenyl)propionic acid being the major catabolite identified (97).

Pellegrini et al. studied the behavior of phenolic compounds from chia (*Salvia hispanica* L.) seeds, as possible ingredients for functional foods, obtaining interesting results. Despite the fact that only a low percentage of phenolics and flavonoids were available for absorption in the intestinal stage, the *in vitro* antioxidant activity decreased after the oral phase and increased after the gastric one, reaching the highest activity after the intestinal stage (98). For the DPPH assay, this behavior has been observed for a high number of polyphenol-containing fruits (98, 99). The ABTS antioxidant activity increased significantly after intestinal digestion, possibly due to the conversion of phytochemicals to more bioactive metabolites and the release of other bioactive species from the matrix. At the end of the digestion simulation, FRAP values were higher than in the case of undigested samples, confirming again this behavioral pattern (98). Balakrishnan and Schneider showed that when there is a much higher content of flavonoids in the matrix, as in the case of quinoa seeds, the total phenolic content is higher after GI digestion simulation. This could suggest that flavonoids show a better release from the matrix after digestion which, in this case, corresponds to the increase of antioxidant activity after every digestive phase. For the flavonoids extracted from quinoa products there was an increase in bioaccessibility and an increase in antioxidant activity for DPPH and ORAC assays (100).

In certain cases, formulating plant material as capsules results in an improved profile of the released polyphenols. Accordingly, Castaldo et al. determined that acid-resistant capsules filled with fennel (*Foeniculum vulgare* Mill.) waste provided protected polyphenols, during duodenal and colonic digestion, in comparison to non-acid-resistant capsules, which ultimately led to a rise in the antioxidant effect detected by DPPH, ABTS and FRAP assays. Even though the antioxidant activity was lower in comparison to the undigested material, the colonic stage led to the highest activity among all the digestive phases. The major compounds detected were 4-caffeoylquinic and 3,4-dicaffeoylquinic acid (26). Another example of the influence of encapsulation on bioactivity was studied by Peanparkdee et al. using Thai rice bran extracts as material. Among all the carriers used for the formulation, gelatin showed the best release of bioactive phytochemicals (phenolic acids, flavonoids, and anthocyanins) and the highest antioxidant activity, in contrast to a gelatin and gum Arabic complex. In this case, the authors suggested that type A gelatin could be used as a protective material to increase the extracts’ bioaccessibility and activity (101). Annunziata et al. studied the influence of gastrointestinal simulation and the antioxidant activity of three sorts of tea (*Camellia sinensis*), formulated as lyophilized powders encapsulated in acid-resistant capsules. In this formulation, the bioaccessibility of the polyphenolic compounds, and, at the same time, the antioxidant activity, were higher in the colonic phase than in the duodenal phase, which highlights the benefic impact of gut microbiota. Thus, using acid-resistant formulations could be a good strategy in nutraceuticals for rising the bioaccessibility of polyphenols, but at the same time supplementary research is needed to understand the mechanism of microbial-mediated release of dietary polyphenols (27).

Singh and Kitts studied the behavior of anthocyanins in tart cherry (*Prunus cerasus* L.) extracts formulated with mineral clay and concluded that there is a high stability of anthocyanins in the gastric phase, but duodenal digestion induces more than 70% of degradation or conversion of the compounds. Interestingly, the antioxidant activity determined using the oxygen radical absorption capacity (ORAC) assay raised almost two-fold after the complete simulation of GI digestion, but the authors suggested this can be caused by the presence of other phytochemicals in the food matrix, which increases bioaccessibility after digestion (28).

## 4. Carotenoids

**Figure 3:**
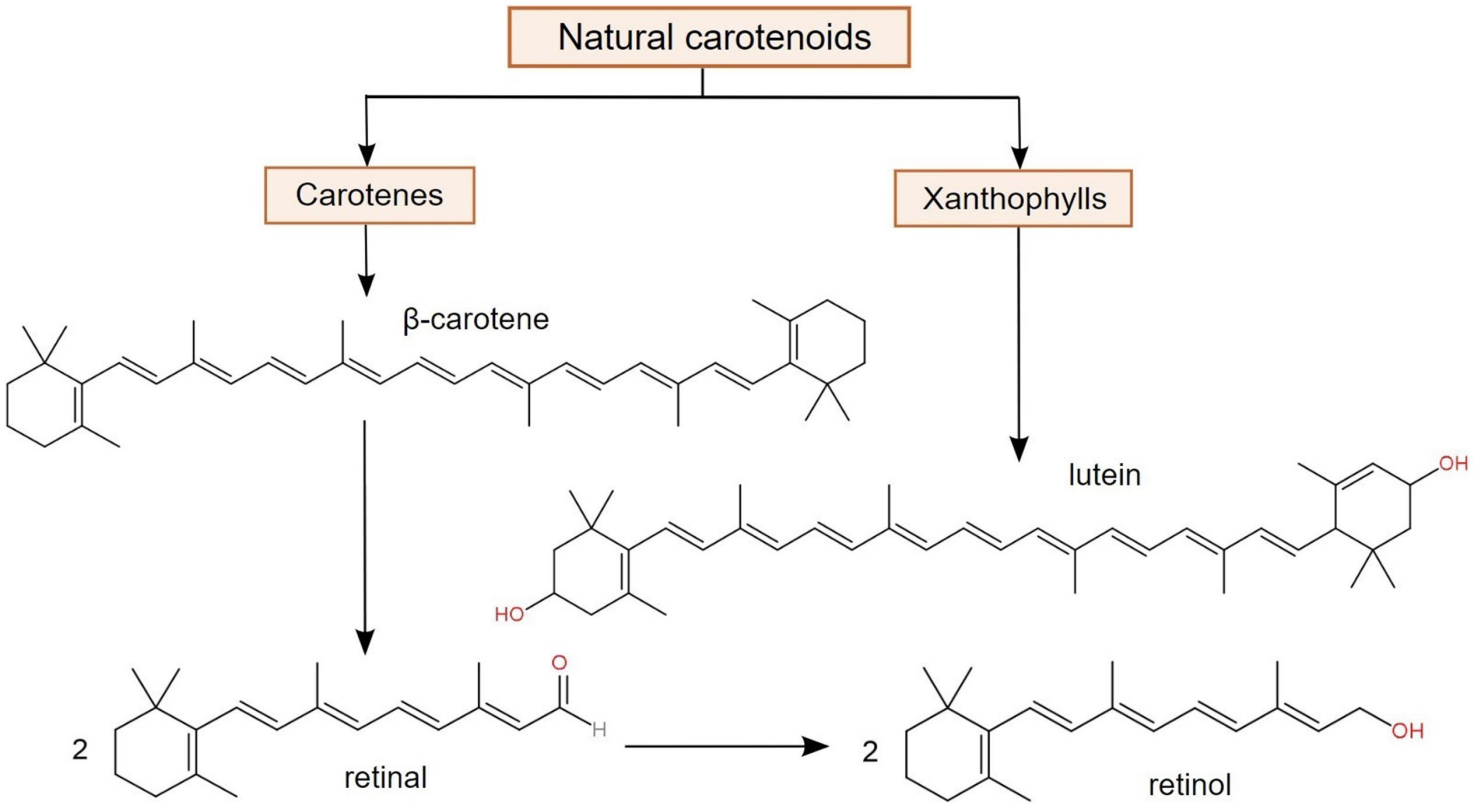
Major classes of carotenoids and the bioconversion of β-carotene into two forms of vitamin A (retinal and retinol), demonstrating its function as provitamin A (103, 104).

Among the natural terpenoid compounds, carotenoids are one of the most important and distributed pigments with significant relevance in certain processes, such as photosynthesis. Even though carotenoids are actively biosynthesized in a variety of organisms, e.g., algae, plants, and prokaryotes, animals, in addition to not being able to produce them, can only obtain them from external sources, such as dietary supplements of fortified foods (102). Structurally, carotenoids are hydrophobic tetraterpene derivatives, containing a chromophore represented by conjugated double bonds. Depending on their chemical nature, there are two major classes of carotenoids, namely carotenes (such as α- and β-carotene), which are hydrocarbons, and xanthophylls (such as lutein and zeaxanthin), which contain oxygenated functional groups. Only carotenes are considered as provitamin A carotenoids, since they represent the only class that is converted to retinal (retinaldehyde), retinol and retinoic acid (12, 103), as suggested in Figure 3.

Even though carotenoids are generally recognized as non-essential phytochemicals, they can lower the risk of chronic diseases, such as coronary heart disease, type 2 diabetes, ophthalmological diseases and even cancer (102). Although certain carotenoids act as molecules with beneficial biological activity (e.g., lutein and zeaxanthin acting as antioxidant and ultraviolet filters), as well as precursors for vitamin A, the overall bioavailability of these compounds is usually problematic. This results from the known lipophilic nature of carotenoids, which in turn indicates a need for a micellization process before absorption. Thus, it can be considered that mixed micelles formation is essential to favor the absorption of these compounds in a satisfactory manner (12, 105).

The bioaccessibility and bioavailability assessment of carotenoids from different types of foods of plant origin has great research value, given that this class of compounds is usually stored in specific organelles, in cells with different cell wall thickness and fibrosity (106). In comparison to the more hydrophilic classes of compounds that are assessed with bioaccessibility studies, certain extra steps are needed for carotenoids, i.e., a combination of centrifugation and filtration, which are necessary to separate the solubilized aqueous phase (which is accessible to enterocyte uptake) from the insoluble matrix (34). Moreover, to address the stability, micellization and possible cellular uptake of carotenoids, bioaccessibility testing can be used alongside with Caco-2 cell cultures, with good applicability for food products and supplements. These cells can incorporate carotenoids that are solubilized in micelles, and can be adapted to evaluate either the cellular uptake or the transepithelial transport of carotenoids (107, 108).

### 4.1. Bioaccessibility of carotenoids from nutraceuticals and functional foods

Given their potential beneficial effect on human health, carotenoids represent one of the most important and increasingly popular classes of nutraceuticals, being frequently integrated into dietary supplements and/or in functional foods. However, as mentioned above, there is a difficulty regarding carotenoids’ bioavailability, which can be explained based on several physiochemical and biochemical factors (9, 102, 109).

The bioaccessibility of compounds with preponderant lipophilic properties is described in a much more complex manner, including the concept of necessary solubilization from the matrix to form the mixed micelles from phospholipids and bile salts, which are a part of the GI fluid, and from monoacylglycerols (9, 15, 110). This micellization process is of utmost importance for the final bioavailability of carotenoids since it functions as a prerequisite for enterocyte uptake and intestinal absorption. Thus, the presence of lipids from dietary sources can enhance carotenoid uptake, yet dietary fibers seem to have opposite effects, possibly by interfering with the generation of mixed micelles (102). Recent studies considered other dietary factors that can influence this bioaccessibility; a great example is the presence of co-digested proteins, which can act as emulsifying agents. The influence of proteins can have a positive impact, by actively promoting the emulsion formation and stability, or negative, by preventing the access of enzymes to lipidic content (102, 111). A recent study on spinach showed that carotenoid bioaccessibility could also get higher as a result of co-ingestion with nanoemulsions as excipient, which again highlights the importance of formulation studies for a better release of phytocompounds from supplements and food matrices (112). The high impact of dietary fat on carotenoid bioaccessibility has been known for a long time. Besides the role in micelle formation, the presence of fat also stimulates bile salts secretion (105).

One of the most important factors linked to carotenoid bioaccessibility is the dosage form. For example, a tablet with no lipidic content other than carotenoids would not be capable of delivering an adequate quantity of these compounds. Nevertheless, certain forms, like gelatin shells filled with carotenoids in an oily medium, can induce a better delivery. The type of oil selected for the formulation is also very relevant, and the bioaccessibility seems to be higher when emulsified oils are chosen (9). At the same time, a several formulation factors can alter the bioaccessibility of carotenoids, such as: the concentration of the oil droplets (113), the emulsifier type (114), the composition of the excipients (for example, the core material, the emulsifiers and the stabilizers), the structure of the formulation (including particle size and charge) and even the physical state of the delivery system (115).

Moreover, different encapsulation methods have the advantage of protecting the compound from degradation, which is prone to happen due to exposure to light, oxygen and temperature. In the case of β-carotene, lipidic microencapsulation is the preferred choice for improving stability, and can be achieved using different forms, including emulsions, nano- and microemulsions, emulsion electrospraying, liposomes, nanoparticles, etc. (115, 116). A special category applicable to carotenoids is represented by Pickering emulsions, which are stabilized by solid particles (including proteins of plant and animal origin) that are absorbed at the interface (117, 118). Carotenoids can also be formulated as gels, for example by combining stabilized emulsions and hydrogels (prepared from starch). One of the advantages of hydrogels lies in their ability to prevent the aggregation of lipid droplets during digestive processes (119). Dispersible phytosterols have been shown to rise the bioaccessibility of carotene, especially mixed with maltodextrin (120). Another significant factor in formulation is the drying process, and suitable techniques should be considered in the case of carotenoids. For example, β-carotene shows high degradation after spray drying, and encapsulation using oil in water emulsion can even lead to complete degradation in the gastric phase (121). In order to predict optimal storage conditions for encapsulated carotenoids, formulation studies should also include modeling, implying models such as Arrhenius, Weibull, Regression, or Higuchi (122).

Fucoxanthin, classified as a “marine carotenoid,” given that it is produced in seaweed and diatoms, is frequently used as an ingredient in nutritional supplements. Extensive research regarding fucoxanthin concluded that it has a low bioaccessibility, however this can be improved by a variety of formulatios, such as: encapsulation in liposomes, micelles, nanogels (including chitosan-glycolipid nanogels), nanoemulsions and chitosan nanoparticles. Another method with noteworthy relevance for functional foods is the development of dairy foods fortified with such carotenoids (123, 124).

In the case of functional foods, the dietary matrix is important because it dictates the way by which the carotenoids will be released from the chemical complexes. Within plant tissues, the majority of carotenoid compounds are concentrated in chromoplasts, either crystalized or solubilized in oil droplets, and these forms are easier to access than other compounds that are usually complexed with proteins. Mechanical disruption of plant material can also influence the bioaccessibility of carotenoids, but this seems to be dependent on the chemical structure (105). In the case of xanthophylls, the majority is found in the form of carotenoid esters in plant material, and these esters need to be deacylated by GIT lipases prior to absorption. However, recent studies suggest that they suffer re-esterification inside the organism (15). Also, nonthermal technologies (such as high hydrostatic pressure, ultrasounds and pulsed electric fields) used in food processing are superior to thermal methods, and are reported to increase carotenoids’ bioaccessibility (125).

### 4.2. Influence of bioaccessibility on the activity of carotenoids

Vulić et al. performed extensive research to assess the link between the simulated digestion and the bioactivity of encapsulated carotenoids isolated from red pepper waste. While concluding there was a rapid initial release of carotenoids from the proteic matrix, there was a slighlty higher bioactivity observed for freeze-drying in comparison to spray-drying. As a formulation method, encapsulation seemed to offer protection against pH changes and the activity of the digestive enzymes, overall rising the bioaccessibility and the bioactivity of the phytocompounds present in the matrix. Morover, the determination of antioxidant activity of carotenoids highlighted the fact that these compounds show higher activity in oil–water emulsions, acting as a protective layer against oxidation (29).

As previously stated, the formulation is highly important for foods fortified with carotenoids or nutraceuticals. Lyu et al. (30) studied the effect of particle size of corn oil in the powders obtained from seed-used pumpkin byproducts, trying to correlate the bioaccessibility with the antioxidant activity of the carotenoids from the products. In the DPPH assay, lutein and β-carotene were responsibile for the majoritiy of the activity and, for FRAP, the highest contribution was due to the content of cryptoxanthin and α-carotene. The study concluded that there is an increase in antioxidant activity as a result of adding corn oil and using powders with smaller particle size. In the case of carotenoids, many studies showed that their release from the food matrix during gastrointestinal digestion results in a typical lower bioaccessibility. Kumari and Gunathilake showed that the carotenoids content (quantified as β-carotene and lycopene) from fresh, dried and juiced black plum (*Syzygium caryophyllatum*) methanolic extracts decreased gradually during the digestion stages, which has been correlated with the decrease in antioxidant activity (as total antioxidant activity and ABTS assay, among others tests) (31).

## 5. Minerals

Minerals are important elements for the human organism since they perform a variety of functions, such as growth, biochemical processes, promoting health, among others. The absorption of minerals after digestion is completed by active and passive transport (6). *In vitro* methods for determining the bioaccessibility of minerals from plant sources have been used. However, these have no power to predict the absorption pattern, which is extremely relevant in this case. To improve these methods, a better estimation by *in vitro* assessment has been achieved using a Caco-2 cell line grown on different supports, which can be applied to a variety of food sources (126, 127). In general, the dialysability assay is a suitable method for determining mineral bioaccessibility. In this method, originally developed for iron bioaccessibility, the measurement of soluble minerals (as proportion of minerals that diffused through a semi-permeable membrane) is done after *in vitro* simulated gastrointestinal digestion, with dialysis being used for adjusting the pH between the gastric and intestinal phases (128, 129). Subsequently, improved bioaccessibility testing methods have been developed, one example being the BARGE method (developed by The Bioaccessibility Research Group of Europe). This method, previously used for the determination of metals in soils and certain foods, can also be successfully utilized for a large variety of metals from nutritional supplements (e.g., Cr, Cu, Fe, Mg, Mn, Mo, Se, and Zn) (130). The protocol uses an initial saliva phase, followed by a simulation of gastric and intestinal compartments (131).

Among functional foods, an interesting category is represented by probiotics, foods containing lactic acid-producing bacteria (such as *Lactobacillus* and *Bifidobacterium*), which can provide health benefits (132). In combination with different types of cheese, probiotics have been observed to enhance the bioaccessibility of minerals (especially magnesium and calcium). At the same time, the colonic microbiota significantly influences the bioaccessibility of zinc from plant matrices since it can reduce the dissolution of this mineral in the colon phase. Organic derivatives of certain minerals show higher bioactivity compared to the simple, inorganic form. For example, selenomethionine shows a much higher bioaccessibility in the presence of probiotic bacteria (e.g., 98% for *Bifidobacterium longum*), since they are able to convert inorganic Se into organic. Overall, mineral-enriched probiotics present good potential for the fortification of functional foods (133). Moreover, functionalization of Se nanoparticles with plant material can rise the bioaccessibility and compatibility of this mineral (134).

Different strategies can be applied to enhance the bioaccessibility of minerals in foods, including their co-administration with plant material. For example, Yun et al. have found that the bioaccessibility of calcium can be improved by adding a *Moringa oleifera* leaf hydrolysate to kimchi. As for functional foods, this type of approach could be used for mineral supplementation (135). Moreover, another study concluded that iron and zinc bioaccessibility was enhanced in the case of pearl millet fortified with roselle calyces and baobab fruit pulp. This could be explained by the presence of high levels of citric and ascorbic acid, which are organic compounds that promote the bioaccessibility of such minerals. The strategy of food-to-food fortification of cereal with other plant sources can serve as a method to improve the bioavailability of certain minerals (136).

Regarding the impact of nutraceutical formulation on the bioaccessibility of minerals, different factors influence this process. In the case of iron, recent studies have shown that microcapsules could serve as a really good formulation, by limiting the interaction with food and by providing protection against oxidation (137). Moreover, different iron salts, like ferric pyrophosphate and ferric ammonium citrate, show a high efficacy when they are encapsulated in liposomes (138). Using the BARGE method, Tokalıoğlu et al. assessed a variety of minerals from nutritional supplements and concluded that Mg, Mo, and Se have similar bioaccessibility in gastric and gastro-intestinal phases, Fe, Mn, and Zn are more bioaccessible from the gastric phase, and that there is a high variability for Cu and Cr bioaccessibility in both phases (130). To conclude, Scrob et al. showed that for several dried fruits, Na, K, Mg, Fe, Mn, and Cu present moderate bioaccessibility, but Zn is not bioaccessible after the simulation (139).

## 6. Vitamins

As components of nutraceuticals, vitamins are essential for both human and veterinary supplementation, because they influence the health, development and growth of the organism, even being required for reproduction (140, 141). Among all the compounds that are vitamins, only 13 are considered to be “true vitamins” and, based on their solubility, they can be further classified in water soluble (B vitamins and vitamin C) and fat or oil soluble (vitamin A, D, E and K). The beneficial effect of vitamins on the human organism is well established, and some notable examples are vitamin D (improving bone resistance), vitamin A (improving visual activity) and vitamin C (acting as a strong natural antioxidant). Their essentiality in such processes dictates the need for an appropriate consumption of nutraceuticals and functional foods, which can help with supplementation (138).

One of the main challenges related to the bioaccessibility of vitamins is their low chemical stability. For this reason, vitamin nutraceuticals might need special formulation techniques. Like carotenoids, hydrophobic vitamins (such as vitamins D and E) require lipid-based nanocarriers (138). At the same time, the bioaccessibility of these vitamins is usually the rate-limiting step for their activity (47). The bioaccessibility of lipid soluble phytocompounds can be changed by a good manipulation of formulation properties. In the case of emulsion-based delivery systems, some factors that can influence the process are: the composition of oil and aqueous phases, the droplet size, aggregation and physical state, and the interfacial properties (142). Another factor that influences the bioaccessibility of vitamins is the presence of minerals, and one of the most well-known examples is the interaction between calcium and vitamin D. A possible mechanism that causes the reduction of lipophilic vitamin bioaccessibility is the perturbation of mixed micelles by precipitation (specifically for divalent minerals) and alteration of zeta potential, lowering their release (138, 143).

In the case of water-soluble vitamins, there can be a significant decrease in bioaccessibility for folate, vitamin C and B_1_, which is explained due to the difference in pH between the gastric and intestinal phases (16). In foodstuffs of plant origin, the bioaccessibility of water-soluble vitamins is relatively low, explained by the presence of dietary fibers, but also by the characteristics of the GIT, such as the temperature and the pH. This has been well established in the case of vitamins B_1_, B_2_ and B_3_ (144).

**Figure 4:**
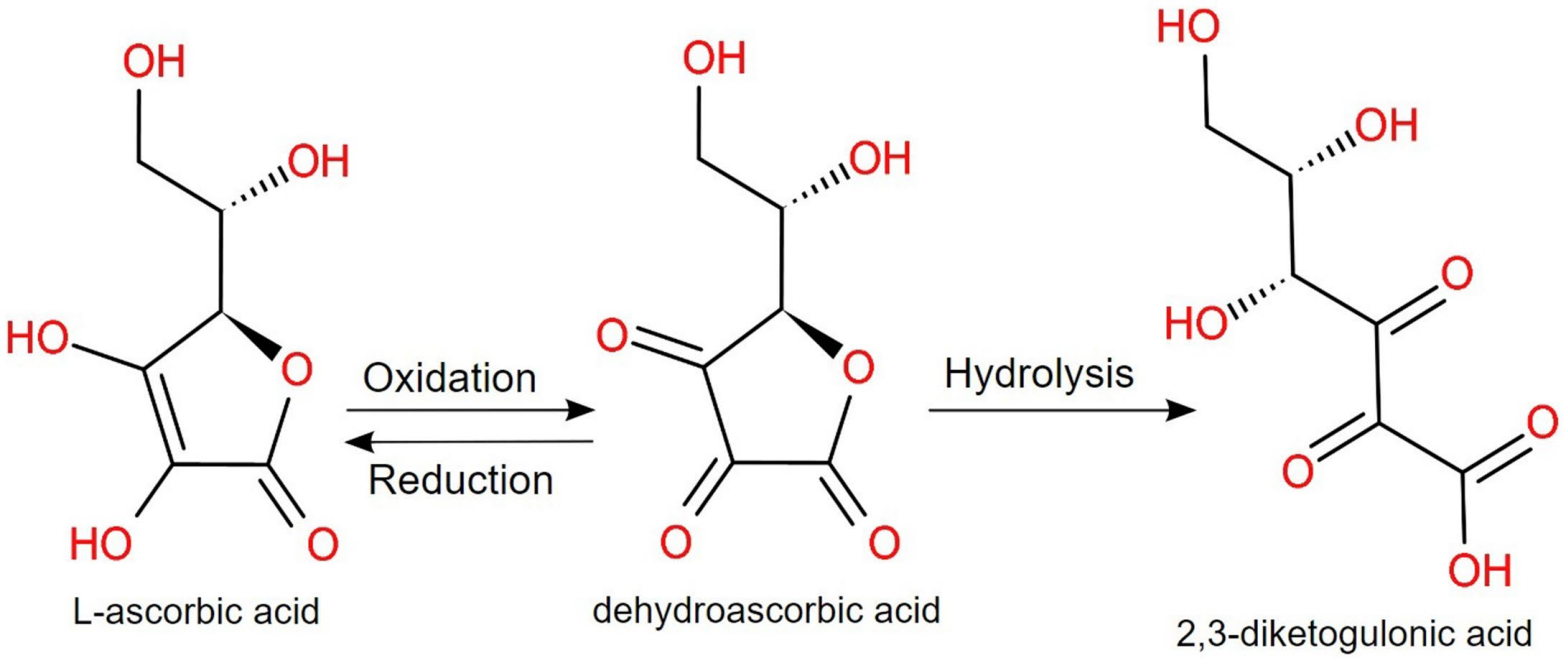
At alkaline pH, L-ascorbic acid (vitamin C) is reversibly oxidized to dehydroascorbic acid, which irreversibly hydrolyzes to 2,3-diketogulonic acid (145).

As for vitamin C (or ascorbic acid), a neutral or alkaline pH induces the oxidation to dehydroascorbic acid, which is converted irreversibly to 2,3-diketogulonic acid (16, 145), as displayed in Figure 4. Brandon et al. investigated the maximum bioaccessibility of vitamins from various products, with interesting conclusions. In the case of dietary supplements and fortified food, folic acid and vitamin C showed a higher bioaccessibility than infant formulas, but for vitamin A, the feeding status, the composition and the encapsulation technique have a higher impact on the bioaccessibility (146). Moreover, in infant foods rich in vitamin C, fortification with fruits and vegetables is important, but additional content of vitamin C in commercial products is needed due to the significant loss that can happen during processing and digestion. This highlights the sensibility of vitamin C under temperature, light and pH variations and, for children, the bioaccessibility could be even lower, due to a higher gastric pH than adults (147). For potential future vitamin C fortified foodstuffs, it is recommended that various factors are investigated, including the presence of flavanone, minerals and other vitamins in the final product (148).

Another essential water-soluble vitamin is vitamin B_12_ (or cobalamin), an example of corrinoid which acts as a common ingredient in fortified foods and supplements. The deficiency of this vitamin can be a cause of megaloblastic anemia and neuropathy, among other conditions (16, 149). In products of plant origin, vitamin B_12_ should be absent, since its biosynthesis is limited to some bacteria and archaea species. Furthermore, foods that are fermented or contaminated with such microorganisms can be a source of vitamin B_12_ (150). Another source of vitamin B_12_ is represented by cyanobacteria (151). Considering the main presence of this vitamin in meat, culinary treatment should be considered in the assessment of bioaccessibility. Recently, Afonso et al. showed that in chub mackerel (*Scomber colias*), vitamin B_12_ bioaccessibility fluctuates between 77 and 83%, displaying seasonal variability (152). A relevant method for the fortification of plant foods is represented by using microorganism produced vitamin B_12_, and in this case a heat treatment would be necessary to release the vitamin from the bacterial cells. The bioaccessibility is different according to the nature of the food product (153). Nevertheless, either from microorganisms (bacteria, such as *Propionibacterium freudenreichii* and *Lactobacillus brevis*, and yeasts) or synthetic origin, vitamin B_12_ is a classic example of nutrient that can be used in food fortification of cereals, as a method to prevent deficiencies (154, 155). As previously stated, nutraceuticals and supplements containing lipophilic vitamins should be carefully formulated to assure a maximal bioaccessibility. However, this is rather difficult to apply since there are a lot of factors that can withhold their proper release from the matrix (138). The quality and the nature of the oil phase have significant importance. Moreover, lipophilic vitamin bioaccessibility seems to be increasing as a result of reducing the oil droplet size in emulsions, probably because of a higher oil–water interfacial area (17). For vitamin D_3_ oil-in-water nanoemulsions, Tan et al. found that the bioaccessibility is higher when using digestible oil (corn oil), since vitamin molecules remain trapped inside the droplets if an indigestible oil phase (mineral oil) is used instead. By using an oil mixture, the bioaccessibility of vitamin D_3_ is intermediate between only digestible and indigestible oil (156). For the same vitamin, Ozturk et al. found that nanoemulsions obtained with oils containing long chain triglycerides (LCT) show maximal bioaccessibility, which highlights the importance of oil composition in the formulation of nutraceuticals with lipophilic nutrients (157). The same phenomenon has been observed for vitamin E (for α-tocopherol acetate, in particular), for which long chain triglycerides emulsions increase the bioaccessibility more than medium chain triglycerides (MCT). The explanation arises from a higher efficacy of mixed micelles to solubilize the vitamin molecules and a higher ability to hydrolyze the acetate derivative to α-tocopherol (158). Regarding the influence of the emulsifier type, Lv et al. (159) showed that animal-based emulsifiers (whey protein isolate) induce a higher bioaccessibility than plant-based ones (gum arabic and quillaja saponin) in the case of vitamin E emulsions. Nonetheless, future studies should include suitable designs, that can clarify the relationship between the *in vivo* functionality and bioaccessibility of lipophilic vitamins, assisted by intensive kinetic data (160). Jensen et al. (161) have assessed the bioaccessibility of different vitamin K vitamers (phylloquinone and menaquinones) from different food matrices, concluding that their bioaccessibility was lower from supplement powder than from canola oil and pasteurized eggs, with broccoli showing the lowest value. The differences might arise from the different food matrix nature and from the high variation in fat content, which is important for the formation of mixed micelles in which vitamin K can solubilize. Newer methods for raising the bioaccessibility of lipophilic vitamins are under development, and the usage of Pickering emulsions seems to be gathering new interest in the development of fortified foods (162). However, the impact of excipients in the formulation must be considered according to the final products’ quality demands.

## 7. Discussion and critical remarks

Bioaccessibility assessment can be performed through *in vitro* methods, in which the physiological conditions inside the GIT are simulated. The main argument in favor of bioaccessibility testing is that it can act as an indicator of maintaining the bioactivity of phytochemicals after all the digestion stages, serving as a good alternative to the laborious and expensive *in vivo* testing. Nutraceuticals and functional foods are used as health-promoting products, and this trend indicates a special need for bioactivity evaluation after exposure to gastrointestinal conditions. There is no universally accepted bioaccessibility determination method, and its assessment should be done considering various influential factors. The variability in the properties of phytochemicals from different structural classes makes this process even more challenging. For nutraceuticals and dietary supplements containing lipophilic compounds (including carotenoids and fat-soluble vitamins), special formulation techniques should be considered to maximize their release from the matrix. Recent studies assessed the bioaccessibility in different nanodelivery and emulsion systems, and the most important enhancing factors seem to be oil phases containing long chain triglycerides and the use of digestible oils rather than indigestible ones. In certain cases, encapsulation is a preferred dosage form, providing necessary protection against degradation.

Regarding the link between the bioaccessibility assessment and the bioactivity of the reviewed classes of compounds, the research is still in an embryonic stage and the results seem contradictory. In the case of polyphenols, the activity after simulated digestion depends strictly on the subtype of the compound, and there are significant differences between simple non-flavonoids, flavonoids, and anthocyanins. Overall, certain formulation techniques (such as encapsulation and freeze-drying) can enhance polyphenols’ bioaccessibility, and recently researched functional foods can be used in the fortification of foodstuffs.

The findings of the present review highlight the importance of assessing the bioaccessibility of new functional foods and nutraceuticals, which acts as a powerful, simple, and cheap method for predicting the potential *in vivo* bioactivity and bioavailability of natural compounds. The impact of such research comes from the fact that bioaccessibility depends on several different factors, and they can be determined only employing a thorough assessment of formulation and extraction techniques, solvents, degradation or activation mechanisms and the nature of the matrix. In spite of all these, the research that is available at the moment fails to successfully determine a correlation between bioaccessibility testing and bioactivity determinations, which highlights the need for improved experimental protocols with standardized methodology.

Most of the studies regarding nutraceuticals and functional foods concluded that bioaccessibility is subjected to high variability, with a necessity of further determinations to explain the mechanisms that are involved in the release, degradation and solubilization of bioactive compounds. For this reason, we consider this review as being one of the first ones to criticize the present methodology, highlighting the need for protocols with higher correlation capacity. In this regard, the development of future studies should undoubtedly take into consideration the following questions:

1. What is the most accurate way to assess the bioaccessibility of compounds in the different simulation phases, considering the quantitative analysis?
2. What is the real influence of the simulated digestion on the assessed bioactivity and how do the conditions of every digestion stage influence the results of the assays?
3. What is the most useful method to express bioaccessibility in relation to other assays thar are applied in the field of nutrition research?
4. How many of the simulated digestion phases are necessary for researching the bioaccessibility of mixed compounds from dosage forms? Nevertheless, what should be changed in the case of fortified foods?
5. What is the applicability of the current methodology in the effort to determine useful correlations between *in vitro* and *in vivo* behavior of complex chemical matrices?

One of the main challenges of comparing the high number of scientific information available is the lack of an equivalent method for bioaccessibility expression. To facilitate standardization and comprehension of the results, the usage of percentages (as BA%) could be the best option, as we have concluded from evaluating the literature data presented in Table 1. As other authors have recently suggested (13, 38, 163–165), in spite of the advances in food science and human nutrition research, further development of methodology is promptly required.

## 8. General conclusions

The determination of bioaccessibility is a rapid and cheap *in vitro* method, acting as an indicator of phytochemicals’ bioactivity preservation after all the digestion phases. Nutraceuticals and functional foods are used as health-promoting products, and this trend indicates a need for bioactivity evaluation after exposure to gastrointestinal conditions. Several factors influencing the bioaccessibility of bioactive compounds (phenolic derivatives, carotenoids, minerals, and vitamins) in the case of nutraceuticals and functional foods have been discussed. Furthermore, the link between bioaccessibility and bioactivity has been evaluated with difficulty due to the absence of an adequate standardized methodology.

The research data regarding nutraceuticals’ and food products’ bioaccessibility is plentiful, however the current methodology is not helpful in assessing the correlation to *in vivo* bioactivity. Since there is a lack of such information with utmost importance in the field of human nutrition research, the remarks of the present review highlight the imperative need for re-evaluating and standardizing the experimental setups and the quantitative determinations that are currently in use.

## Author contributions

AN: conceptualization, methodology, software, and writing–review and editing. MB: writing–review and editing. LB: visualization, writing–review and editing, and funding acquisition. GR: supervision and writing–review and editing. LL: software, methodology, and supervision. CT: supervision and funding acquisition. AM: conceptualization, methodology, and funding acquisition. CB: methodology and supervision. GC: supervision. All authors have read and agreed to the published version of the manuscript.

## Funding

This work was supported by a grant of the Romanian Ministry of Education and Research, CNCS–UEFISCDI, project number PN-III-P2-2.1-PED-2019–5360.

## Conflict of interest

The authors declare that the research was conducted in the absence of any commercial or financial relationships that could be construed as a potential conflict of interest.

## Publisher’s note

All claims expressed in this article are solely those of the authors and do not necessarily represent those of their affiliated organizations, or those of the publisher, the editors and the reviewers. Any product that may be evaluated in this article, or claim that may be made by its manufacturer, is not guaranteed or endorsed by the publisher.
